# Tumor-infiltrating lymphocytes predict response to anthracycline-based chemotherapy in estrogen receptor-negative breast cancer

**DOI:** 10.1186/bcr3072

**Published:** 2011-12-08

**Authors:** Nathan R West, Katy Milne, Pauline T Truong, Nicol Macpherson, Brad H Nelson, Peter H Watson

**Affiliations:** 1 Trev & Joyce Deeley Research Centre, British Columbia Cancer Agency, 2410 Lee Ave., Victoria, British Columbia, V8R 6V5, Canada; 2 Department of Biochemistry and Microbiology, University of Victoria, PO Box 3055, STN CSC, Victoria, British Columbia, V8W 3P6, Canada; 3 Department of Radiation Oncology, British Columbia Cancer Agency Vancouver Island Centre, 2410 Lee Avenue, Victoria, British Columbia, V8R 6V5, Canada; 4 Department of Medical Oncology, British Columbia Cancer Agency Vancouver Island Centre, 2410 Lee Avenue, Victoria, British Columbia, V8R 6V5, Canada; 5 Department of Medical Genetics, University of British Columbia, 4500 Oak St. Unit C201, Vancouver, British Columbia, V6H 3N1, Canada; 6 Department of Biology, University of Victoria, PO Box 3020, Station CSC, Victoria, British Columbia, V8W 3N5, Canada; 7 Department of Pathology and Laboratory Medicine, University of British Columbia, Rm. G227 - 2211, Wesbrook Mall, Vancouver, British Columbia, Canada

## Abstract

**Introduction:**

Infiltration of breast tumors by tumor-infiltrating lymphocytes (TIL) has been associated with sensitivity to anthracycline-based chemotherapy. However, it is unclear whether this is true within the estrogen receptor-alpha (ER)-negative subset of breast tumors that frequently manifest high TIL levels.

**Methods:**

The association of TIL with short-term and long-term clinical response to anthracycline-based therapy was assessed in two independent ER-negative breast cancer cohorts in which patients were categorized as TIL-high or TIL-low. We defined an eight-gene lymphocyte mRNA expression signature (including *CD19*, *CD3D*, *CD48*, *GZMB*, *LCK*, *MS4A1*, *PRF1*, and *SELL*) and used unsupervised hierarchical clustering to examine the association between TIL and short-term response to neoadjuvant chemotherapy in a previously published cohort of ER-negative tumors (*n *= 113). We also examined the association between TIL and long-term chemotherapeutic efficacy in a second cohort of ER-negative tumors (*n *= 255) with longer than 6 years of median follow-up by using tissue microarrays and immunohistochemistry (IHC) for detection of CD3, CD8, CD4, CD20, and TIA-1.

**Results:**

In patients with ER-negative tumors treated with neoadjuvant anthracycline-based chemotherapy, pathologic complete responses (pCRs) were achieved by 23 (74%) of 31 TIL-high patients and 25 (31%) of 80 TIL-low patients (odds ratio (OR), 6.33; 95% confidence interval (CI), 2.49 to 16.08; *P *< 0.0001). Multivariate logistic regression with standard clinicopathologic features demonstrated that only tumor size (*P *= 0.037) and TIL status (*P *= 0.001) were independent predictors of anthracycline response. In the second cohort, adjuvant anthracycline-based therapy was associated with increased disease-free survival (DFS) only in patients with high levels of intraepithelial CD3^+ ^TIL (*P *= 0.0023). In contrast, outcomes after CMF treatment (cyclophosphamide, methotrexate, and fluorouracil) showed no association with CD3 status. In both cohorts, cytotoxic T-cells were the primary TIL subtype associated with anthracycline sensitivity. Finally, TIL significantly predicted anthracycline sensitivity for both the Her2-positive and triple-negative tumor phenotypes.

**Conclusions:**

ER-negative breast cancers with high levels of TIL have heightened sensitivity to anthracycline-based chemotherapy, as assessed by the immediate response to neoadjuvant therapy and long-term outcome following adjuvant therapy. Investigations of TIL-based predictive tests to identify patients likely to benefit from anthracycline-based treatments are warranted.

## Introduction

The use of adjuvant systemic therapy for breast cancer has increased substantially in the last few decades, and is now a mainstay modality in clinics worldwide. Although adjuvant chemotherapy has significantly improved patient management, a fundamental limitation of this approach is that many patients fail to benefit from therapy, and physicians are frequently unable to predict the responses of individual patients to a given regimen. As such, much effort has been directed in recent years toward identifying clinical and biological predictive features to better tailor therapy to the needs of individual patients.

Anthracycline-based chemotherapy (regimens involving anthracyclines such as doxorubicin or epirubicin) has been used clinically for more than two decades and has largely supplanted first-generation regimens such as CMF [1]. Anthracyclines are thought to exert their effects through a variety of mechanisms including intercalation of DNA, cross-linking of DNA to proteins, and generation of free radicals [2]. Nevertheless, the precise mechanisms by which anthracyclines exert their therapeutic effects *in vivo *remain unclear, nor has significant progress been made in establishing predictive biomarkers to identify patients likely to derive benefit from anthracycline-based therapy [2,3]. For example, aberrations of *HER2 *and *TOP2A *initially showed promise as anthracycline-response predictors, but have failed to demonstrate consistent clinical utility [2].

Attention has focused recently on the prognostic and predictive potential of antitumor immune responses, detected indirectly via gene expression signatures derived from tumor-infiltrating lymphocytes (TIL) or directly via immunohistochemical TIL staining. In breast cancer, TIL have been shown in several studies to correlate with favorable long-term prognosis, although primarily for hormone receptor-negative, Her2-positive, or high grade/highly proliferative lesions [4-10]. Denkert *et al. *[11] recently reported that TIL were associated with a favorable response to neoadjuvant anthracycline/taxane therapy in two large breast cancer cohorts, providing compelling evidence that TIL could potentially serve as predictive markers for anthracycline-based therapy. However, no such studies have been conducted specifically with the ER-negative breast cancer subset. This is important for two reasons: first, pathologic complete response to neoadjuvant chemotherapy, defined as the complete absence of invasive tumor cells in the breast and lymph nodes after treatment, occurs almost exclusively in tumors negative for ER [12-15]; second, ER-negative tumors typically feature higher levels of TIL than do ER-positive tumors [6,11,16-18]. Therefore, our objective in this study was to determine whether TIL correlate with sensitivity to anthracycline-based chemotherapy in ER-negative breast cancer. To do so, we examined two independent cohorts of ER-negative breast cancer cases. One cohort received anthracycline-based therapy in the neoadjuvant setting, permitting the assessment of short-term clinical responses to therapy. The second cohort included patients with long-term follow-up treated with anthracyclines in the adjuvant setting, allowing us to examine overall and disease-free survival rates after systemic therapy.

## Materials and methods

### Neoadjuvant chemotherapy cohort

To assess the relationship between TIL and short-term response to chemotherapy, we analyzed publically available gene expression data [19] derived from pretreatment incisional or core tumor biopsies from a cohort of patients with ER-negative invasive ductal breast carcinomas. Note that the original publication reporting analysis of gene signatures and treatment response [19] has since been retracted because of concerns regarding the statistical methods used to generate predictive models (see *Lancet Oncol *2011, Feb; 12:116 and *Lancet Oncol *2010 Sep; 11:813-814). However, our analysis is based on original data obtained from the Gene Expression Omnibus website (http://www.ncbi.nlm.nih.gov/geo; accession number GSE6861) and is therefore not affected by this issue. Clinical characteristics of the study cohort are summarized in Table 1. All patients were enrolled in the EORTC 10994/BIG 00-01 clinical trial and received chemotherapy with either FEC (fluorouracil, epirubicin, and cyclophosphamide for six cycles) or TET (three cycles of docetaxel followed by three cycles of docetaxel plus epirubicin) as primary (neoadjuvant) therapy. The primary end point was pathologic complete response (pCR) versus residual disease (RD).

**Table 1:** Characteristics of all assessed patients in the EORTC and MBTB cohorts

		EORTC (*n *= 113)		MBTB (*n *= 255)	
	**Case status**	**Number**	**%**	**Number**	**%**

					
Age at diagnosis	<50 years	56	50%	85	33%
	≥50 years	57	50%	168	66%
					
Tumor size	≤2 cm	3	3%	51	20%
	<2 cm to ≤5	68	60%	142	56%
	>5 cm	42	37%	39	15%
	Unknown	0	0	23	9%
					
Nodal status	Negative	42	37%	88	35%
	Positive	71	63%	142	56%
	Unknown	0	0	25	10%
					
Grade	1-2	36	32%	120	47%
	3	65	58%	119	47%
	Unknown	12	11%	16	6%
					
ER^a^	Negative	113	100%	255	100%
	Positive	0	0	0	0
					
PR^b^	Negative	110	97%	212	84%
	Positive	3	3%	41	16%
	Unknown	0	0	2	<1%
					
Her2^c^	Negative	83	73%	157	62%
	Positive	30	27%	58	23%
	Unknown	0	0	40	16%

Assessed cases were all invasive ductal carcinomas. ^a^ER-negative status defined as <10% of tumor cells stained positive for ER by IHC (EORTC) or <10 fmol/mg protein (ligand-binding assay; MBTB); ^b^PR-negative status defined as <10% of tumor cells stained positive for PR by IHC (EORTC) or ≤15 fmol/mg protein (ligand-binding assay; MBTB); ^c^Her2-positive status inferred based on microarray data (EORTC) or determined by an IHC score of 3 (MBTB).

### Analysis of microarray data

To determine whether lymphocyte-associated gene expression correlated with pCR rates, we analyzed microarray data from the neoadjuvant (EORTC) cohort and obtained an eight-gene TIL signature by using the following steps. We first compiled a list of genes with known biological functions that are expressed at high levels by lymphocytes, based on information in the BioGPS gene portal (http://biogps.gnf.org). Expression data for these genes were compiled and z-normalized (that is, number of standard deviations from the mean). For genes with redundant probe sets, only those probes with the highest interquartile range were selected. To enrich for genes with expression patterns consistent with a lymphocyte cell of origin, expression values of all genes were Spearman correlated with those of *CD247 *(CD3-zeta chain) and *MS4A1 *(CD20), which were chosen as specific and prototypical T- and B-lymphocyte genes, respectively. Only genes that correlated to *CD247 *or *MS4A1 *with a Spearman *R *value of 0.7 or greater were retained, resulting in a list of 33 genes. With this gene set, cases were grouped by unsupervised hierarchical clustering using the centroid method and euclidean distance as the similarity metric (performed with Cluster 3.0). Heat maps and cluster dendograms were produced using Java TreeView.

We further refined the TIL signature by assessing differential expression of each gene in pCR versus RD cases and selecting genes with a Mann-Whitney *t *test *P *value less than 0.05. The final eight-gene signature included *CD19*, *CD3D*, *CD48*, *GZMB*, *LCK*, *MS4A1*, *PRF1*, and *SELL*. Data corresponding to this signature were used to cluster the EORTC cases, as described above. Two cases that failed to cluster with any of the primary centroids were considered outliers and discarded from further analysis, leaving a final assessed cohort size of 111 cases. Univariate associations of TIL status and pathologic parameters with pCR rates were calculated using two-sided Fisher's exact tests in Prism 5.0 (GraphPad, La Jolla, CA). Multivariate logistic regression models for prediction of pCR were constructed in SPSS Statistics 14 (SPSS, Chicago, IL).

### Adjuvant chemotherapy cohort

To assess the relationship between TIL and long-term outcome after adjuvant chemotherapy, we analyzed TIL in primary tumor biopsies or resections taken before adjuvant therapy from a cohort of patients with ER-negative invasive ductal breast carcinomas registered by the Manitoba Breast Tumor Bank (MBTB). Cases were accrued between the years 1988 and 2000 and had a median follow-up time of 83 months. Clinical characteristics of the study cohort are summarized in Table 1 and Additional file 1 (Supplementary Table S1). All selected cases were determined to be ER-negative by a single central provincial clinical laboratory and subsequently managed as ER-negative cases at a single provincial cancer center, where adjuvant systemic therapy was administered to 61% of the cohort. Of these patients, 58% were treated with CMF (cyclophosphamide, methotrexate, fluorouracil), and 37% were treated with one of several anthracycline-based regimens (for example, AC, CAF, CEF, where A is Adriamycin/doxorubicin, and E is epirubicin). Disease-free survival (DFS) was defined as the time from surgery to the first instance of disease recurrence or disease-specific death, and overall survival as the time to death from all causes. The MBTB has approval from the Research Ethics Board, Faculty of Medicine, University of Manitoba, to collect, store, and distribute anonymous cases from its archive under the Canadian Tri-Council Policy Statement waiver of consent. The current study was conducted with approval from the University of British Columbia/British Columbia Cancer Agency Research Ethics Board.

### Tissue microarray construction

An initial cohort of 255 ER-negative cases was selected on the basis of (a) ER-negative status defined by ligand-binding analysis of <10 fmol/mg protein; (b) invasive ductal carcinoma components occupying more than 20% of the tumor section, and (c) no prior therapy. To construct a tissue microarray (TMA), all cases were rereviewed to confirm and select areas for coring of corresponding blocks. Duplicate tissue cores (0.6 mm diameter) were taken from central cellular areas of each tumor with a tissue arrayer instrument (Beecher Instruments, Silver Spring, MD). Prior use and exhaustion of some tissue cores reduced the interpretable cohort size to approximately 160, depending on the TIL marker analyzed.

### Immunohistochemistry and TMA scoring

Immunohistochemistry (IHC) was performed for CD3, CD8, CD4, CD20, and TIA-1 on deparaffinized sections from TMAs by using a Ventana Discovery XT autostainer (Ventana, Tucson, AZ). Ventana's standard CC1 protocol was used for antigen retrieval. Primary antibodies used are as follows (clone, supplier, animal source, concentration): CD3 (RM-9107, Lab Vision, rabbit, 1/150); CD8 (RM-9116, Lab Vision, rabbit, 1/100); CD4 (MS-1528, Lab Vision, mouse, 1/10); CD20 (polyclonal, catalogue no. RB-9013, Lab Vision, rabbit, 1/250); TIA-1 (TIA-1, Abcam, mouse, 1/50). TMA sections were incubated with primary antibodies for 60 minutes at room temperature followed by the appropriate cross-adsorbed, biotinylated secondary antibody (Jackson Immunoresearch, West Grove, PA) for 32 minutes. Antibodies were detected using the DABMap kit (Ventana). Slides were counterstained with hematoxylin (Ventana) and coverslipped manually with Cytoseal-60 (Richard Allan, Kalamazoo, MI).

IHC scoring was undertaken by using a microscope eyepiece grid to standardize the assessed area. In brief, duplicate cores of each immunostained tumor were reviewed at low magnification, and the core exhibiting a tumor/stroma ratio closest to 50:50 and the highest density of positive cells was selected. This core was then assessed at higher magnification (×20 objective) with a grid overlaid on the center of the core. Under a ×20 objective magnification, this grid defined an area of 0.56 mm^2^. The number of positive intraepithelial lymphocytes was quantified within the area of the grid (intraepithelial localization was defined as lymphocytes within tumor cell nests or in direct contact with tumor cells, consistent with the method used by Denkert *et al. *[11]). To account for variation in epithelial-stromal proportions between different samples, intraepithelial TIL levels were calculated by dividing the number of observed intraepithelial TIL by the fraction of grid area occupied by epithelium.

### Statistical analysis of IHC data

All analyses were performed using Prism 5.0. Unless otherwise specified, median values for TIL markers were used as predetermined cut-points to define high versus low cases. Associations of TIL with pathologic features were evaluated with Fisher's exact test. Survival outcomes were assessed via Kaplan-Meier methods and compared using Log-rank tests. All statistical tests were two-sided, with significance established at *P *values less than 0.05.

### Additional validation datasets

We assessed publically available gene-expression data from ER-negative breast cancers within three additional cohorts (accessible via the Gene Expression Omnibus website), the characteristics of which are as follows: GSE21974, a cohort of 32 breast cancer patients (14 ER-negative) treated with neoadjuvant epirubicin, cyclophosphamide, and docetaxel (not previously published); GSE19615 [20], a cohort of 115 breast cancers, 36 of which were ER-negative and treated with adjuvant anthracycline-based therapy (primarily AC and AC plus taxol); and GSE18864 [21], a cohort of 28 triple-negative breast cancers (24 with complete data) treated with neoadjuvant cisplatin. We tested the eight-gene TIL signature in these cohorts by extracting expression data for the eight genes and processing it as described above. In each cohort, patients were divided into two groups based on clinical outcome. For GSE21974, this was based on pathologic complete response or residual disease; for GSE19615, patients were divided based on the presence or absence of distant recurrence after 36 months of follow-up; and for GSE18864, patients were divided into good and poor response groups based on Miller-Payne scores of 3, 4, or 5 versus scores of 0, 1, or 2, respectively. For each response group, the average expression level of each gene was calculated and plotted as a box-and-whiskers plot; Mann-Whitney *t *tests were used to assess differences in overall TIL-signature expression.

## Results

### TIL-enriched tumors are highly sensitive to neoadjuvant anthracycline-based therapy

**Figure 1:**
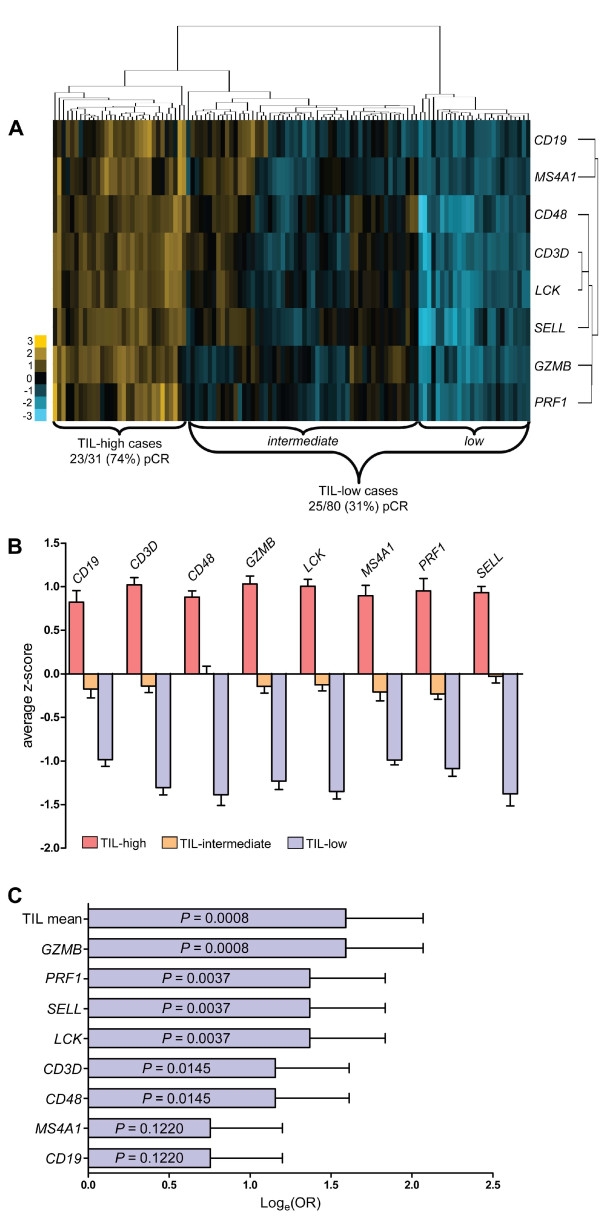
**An eight-gene tumor infiltrating lymphocyte (TIL) signature is associated with pathologic complete response (pCR) after neoadjuvant chemotherapy in the EORTC cohort**. **(a) **Heat map displaying gene (y-axis) and case clusters (x-axis). The TIL-high and TIL-low groups with respective rates of pCR are indicated. Colors correspond to z-normalized expression levels. **(b) **Average expression levels ±SEM of each gene in the three primary centroids. **(c) **Odds ratios (log_e _transformed, ±SEM) for probability of pCR derived from Fisher's exact tests for each gene in the TIL signature. The TIL mean represents average expression levels across all eight genes. High and low gene expression categories were assigned using the upper quartile as the cut-point.

Clustering analysis of the neoadjuvant (EORTC) cohort using an initial set of 33 lymphocyte-associated genes revealed that the combined pCR rate of the two patient clusters with the highest levels of TIL-gene expression was significantly higher than that of the remainder (*P *= 0.0028, Fisher's exact test). This gene list was further refined to derive an eight-gene TIL signature including *CD19*, *CD3D*, *CD48*, *GZMB*, *LCK*, *MS4A1*, *PRF1*, and *SELL *(see Methods), that was used in subsequent analyses. Unsupervised clustering of invasive ductal cases yielded three primary centroids based on relative expression of the TIL signature, as follows: (a) highly enriched for the eight TIL genes, (b) intermediate levels of expression, and (c) low TIL levels (Figure 1a). Cases in the TIL-high cluster generally had expression levels of 0.5 to 1 standard deviations greater than the mean for each gene (Figure 1b). The respective pCR frequencies of the three clusters were as follows: 23/31 (74.2%), 16/54 (29.6%), and 9/26 (34.6%). Because the pCR rates of the TIL-low and intermediate clusters were not significantly different (*P *= 0.80), we treated them as a single TIL-low cluster in subsequent analyses. TIL status showed no significant association with clinical parameters including tumor size, tumor grade, nodal status, patient age, Her2 and PR status, and treatment regimen. The difference in pCR rates between TIL-high and TIL-low patients was highly significant in univariate analysis (*P *< 0.0001; OR, 6.325; 95% CI, 2.487 to 16.08; Table 2). The positive and negative predictive values for TIL status were 74.2% and 68.8%, respectively, with an overall accuracy of 70.3%. TIL status was associated with treatment response to a similar extent in patients treated with FEC (*P *= 0.0035) and those treated with TET (*P *= 0.0059; Table 2). In multivariate logistic regression analysis, the only standard clinical parameter with independent predictive significance was tumor size (Table 3; *P *= 0.037). Notably, TIL status maintained strong significance in this analysis (*P *= 0.001; OR, 6.42; 95% CI, 2.08 to 19.8), demonstrating independence from standard pathologic parameters.

**Table 2:** Two-sided Fisher's exact tests for association of clinical variables and TIL-signature status with probability of pathological complete response (pCR) in the EORTC cohort

	Total (*n *= 111)		FEC treated (*n *= 54)		TET treated (*n *= 57)	
	**OR (95% CI)**	***P *value**	**OR (95% CI)**	***P *value**	**OR (95% CI)**	***P *value**

Size T3 vs T1/T2	0.39 (0.17-0.89)	0.0292	0.60 (0.19-1.87)	0.4103	0.25 (0.19-1.87)	0.0278
Grade 3 vs 1/2	2.80 (1.19-6.61)	0.0226	2.35 (0.73-7.56)	0.1684	2.00 (0.58-6.86)	0.3681
Node 1 vs 0^a^	0.82 (0.38-1.78)	0.6927	1.05 (0.34-3.24)	1.0000	0.65 (0.22-1.92)	0.5823
Age ≥50 vs <50	0.89 (0.42-1.89)	0.8913	1.29 (0.43-3.82)	0.7827	0.62 (0.22-1.77)	0.4311
Her2 A vs N^b^	1.21 (0.52-2.81)	0.6722	2.03 (0.53-7.72)	0.3243	0.81 (0.27-2.46)	0.7824
TET vs FEC	1.22 (0.57-2.59)	0.7022	NA	NA	NA	NA
TIL high vs low	6.33 (2.49-16.08)	<0.0001	6.48 (1.82-23.10)	0.0035	6.84 (1.65-28.39)	0.0059

^a^1, lymph node positive; 0, lymph node negative. ^b^A, amplified; N, not amplified.

**Table 3:** Logistic regression model for prediction of pathologic complete response (pCR) in the EORTC cohort (*n *= 99)

	OR (95% CI)	*P *value
Size T3 vs T1/T2	0.38 (0.15-0.95)	0.037
Grade 3 vs 1/2	1.77 (0.76-4.11)	0.186
Node 1 vs 0^a^	0.62 (0.25-1.51)	0.620
Age ≥50 vs <50	0.69 (0.27-1.75)	0.433
Her2 A vs N^b^	1.97 (0.66-5.92)	0.227
FEC vs TET	0.64 (0.26-1.59)	0.337
TIL high vs low	6.42 (2.08-19.83)	0.001

^a^1, lymph node positive; 0, lymph node negative; ^b^A, amplified; N, not amplified.

The eight genes of our TIL signature were selected because they are expressed primarily by T and B lymphocytes. As shown in Figure 1a, the genes clustered into separate B-cell (*CD19 *and *MS4A1*) and T-cell groups (*CD3D*, *LCK*, *CD48*, *SELL*, *GZMB*, *PRF1*). To determine the relative importance of each gene in the signature, we individually assessed their associations with outcome by comparing pCR rates in cases with expression levels in the upper quartile with those of cases at or below this cut-point (Figure 1c). Intriguingly, both B-cell genes failed to individually predict pCR (*P *> 0.1), whereas all T-cell genes reached statistical significance. *GZMB*, a marker of cytotoxic T lymphocytes (CTL), was the strongest single predictor of pCR (*P *= 0.0008), performing equivalently to the mean expression level of all eight genes.

We further assessed the association of the eight-gene TIL signature with response to anthracycline-based therapy in two small independent cohorts of ER-negative breast cancer with publically available gene-expression and clinical outcomes data (see Methods for details and Additional file 1: Supplementary Figure S1). One cohort consisted of both pre-chemo biopsies and post-chemo surgical specimens; in this group, the samples from complete clinical responders (both before and after chemotherapy) featured significantly higher levels of TIL-signature expression than did those with residual disease (*P *< 0.001). The second cohort consisted of patients treated with anthracycline-based therapy in the adjuvant setting. When patients were categorized based on the presence or absence of distant metastasis after 36 months of follow-up, TIL-signature expression was significantly elevated in disease-free cases (*P *< 0.001).

To determine the association of the eight-gene TIL signature with outcome in a distinct treatment setting, we performed the same analysis on a cohort of patients treated with neoadjuvant cisplatin therapy (Figure S1). In contrast with the anthracycline-based cohorts described thus far, TIL-signature expression was not elevated in patients with favorable responses to cisplatin therapy, suggesting that the eight-gene signature may be associated with clinical response in a regimen-specific manner.

### Tumor-infiltrating T-cells are associated with disease-free survival in patients treated with adjuvant anthracycline-based therapy

**Figure 2:**
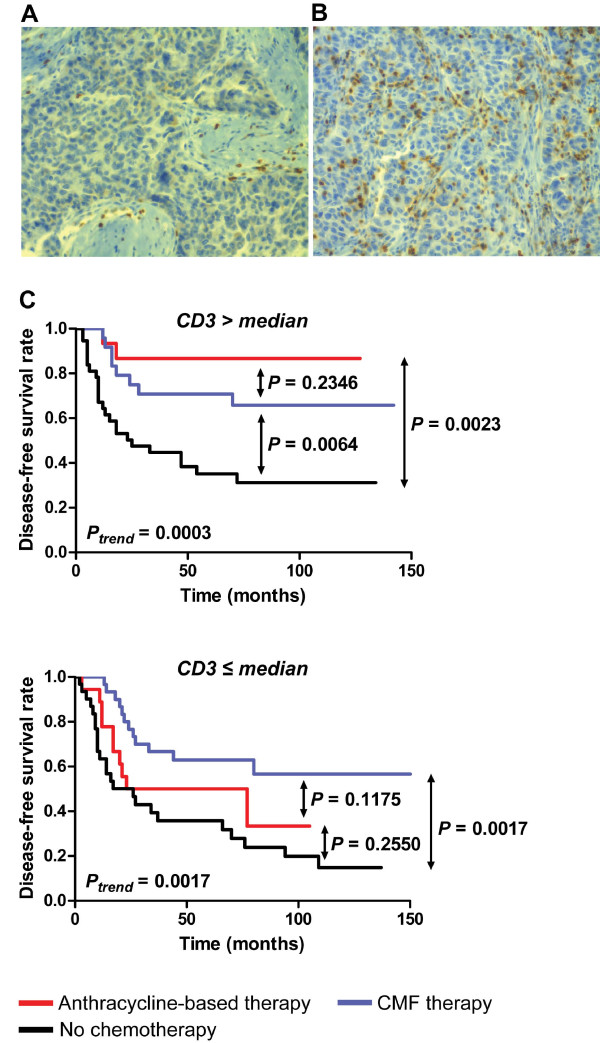
**Long term outcome of patients according to adjuvant chemotherapy and CD3 status**. Representative immunohistochemistry (IHC) staining of CD3 (×40 objective) demonstrating **(a) **low and **(b) **dense intraepithelial infiltration of CD3^+ ^TIL. **(c) **Disease-free survival (DFS) of patients treated with anthracycline-based regimens or CMF (cyclophosphamide, methotrexate, fluorouracil) relative to systemically untreated patients in CD3-high (*n *= 77) and -low (*n *= 79) subgroups. *P_trend_*, log-rank test for trend.

Having found an association of TIL with short-term outcome (pCR assessed after six cycles of chemotherapy) in a cohort of patients receiving neoadjuvant anthracycline-based therapy, we further explored this relationship in a cohort of patients with invasive ductal carcinoma who had been postsurgically treated with adjuvant chemotherapy and for whom long-term outcome data were available (MBTB cohort). We used IHC to detect and quantify TIL expressing CD3 (T-cells) and categorized patients into CD3-high and -low groups by using the population median (six) as a cut-point (Figure 2a, 2b). Within the anthracycline-treated subset of patients, CD3-high status was significantly associated with increased DFS (HR, 0.24; 95% CI, 0.08 to 0.77; *P *= 0.0160). High levels of CD3^+ ^TIL were not associated with patient age, tumor size, or nodal status, but were associated with Her2-negative (*P *= 0.0183) and PR-negative (*P *= 0.0137) status, and strongly associated with high tumor grade (*P *< 0.0001; Table 4). In agreement with our findings from the EORTC cohort, anthracycline-based therapy increased both DFS (HR, 0.28; 95% CI, 0.12 to 0.63; *P *= 0.0023) and overall survival (HR, 0.28; 95% CI, 0.13 to 0.61; *P *= 0.0014) for CD3-high patients, but did not significantly improve survival for CD3-low patients (DFS HR, 0.66; 95% CI, 0.33 to 1.35; *P *= 0.2550; Figure 2c). The corresponding 5-year DFS rates within the CD3-high group were 35.1% for patients who did not receive systemic therapy and 86.7% for anthracycline-treated patients (Fisher's exact test: *P *= 0.0016; OR, 12.00; 95% CI, 2.34 to 61.55). In contrast, the DFS rates of CD3-low patients were not significantly different between untreated and anthracycline-treated groups (35.8% and 50.0%, respectively; Fisher's exact test, *P *= 0.3754; OR, 1.82; 95% CI, 0.56 to 5.93).

**Table 4:** Two-sided Fisher's exact tests for association of CD3 status^a ^and clinical parameters in the MBTB cohort

		CD3 low	CD3 high	*P *value
Age at diagnosis	<50	30	28	0.7442
	≥50	50	53	
Tumor size	T1-T2	60	61	1.0000
	T3	15	15	
Nodal status	Negative	28	48	0.6128
	Positive	48	41	
Grade	1-2	43	19	<0.0001
	3	31	58	
Her2 IHC	0-2	46	63	0.0183
	3	27	15	
PR^b^	Negative	63	75	0.0137
	Positive	17	6	

^a^CD3 high, >median level of CD3^+ ^cells per sample; ^b^ligand-binding assay; negative, ≤15 fmol/mg protein

CMF chemotherapy was administered to approximately one third of the patients in this cohort. Unlike anthracycline-based therapy, CMF effectively increased survival rates for both CD3-high (HR, 0.37; 95% CI, 0.18 to 0.76; *P *= 0.0064) and CD3-low patients (HR, 0.34; 95% CI, 0.17 to 0.67; *P *= 0.0017; Figure 2c). Because no significant clinical differences were found between anthracycline- and CMF-treated patients in this cohort, this implies that CD3 status is predictive of treatment response specifically for anthracycline-based regimens. Although CD3-high, anthracycline-treated patients had a higher 5-year DFS rate than did the CMF-treated patients (86.7% and 70.8%, respectively), this did not reach statistical significance.

### Cytotoxic T-cells are the primary TIL subtype associated with anthracycline sensitivity

**Figure 3:**
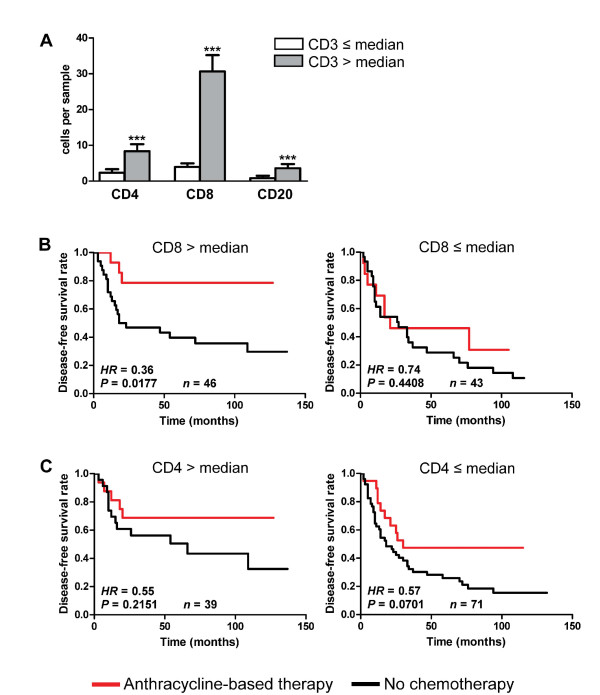
**Additional TIL subsets and their relationship with CD3 status and anthracycline sensitivity**. **(a) **TIL levels in CD3-high and -low cases. Data represent average numbers of TIL expressing CD8, CD4, or CD20 ±SEM. ***P < 0.0001, Mann-Whitney *t *test. **(b) **Association of CD8^+ ^and **(c) **CD4^+ ^TIL with response to anthracycline therapy. Patients were categorized according to levels of CD8^+ ^and CD4^+ ^TIL and assessed for disease-free survival (DFS) with respect to anthracycline-based therapy.

In addition to CD3^+ ^T-cells, we also assessed CD20^+ ^TIL (B-cells) in the adjuvant cohort. Consistent with our results from the neoadjuvant cohort, anthracycline therapy was somewhat more effective for CD20-high than for CD20-low patients, but this did not reach statistical significance (data not shown), indicating that T-cells are the more relevant TIL subset. CD3 staining detects both CD8^+ ^(cytotoxic) and CD4^+ ^(helper) T-cell populations; as such, we further assessed these subpopulations to determine their relative contributions to the correlation of CD3 with treatment response. As expected, CD3 levels were strongly correlated with CD4^+ ^and CD8^+ ^TIL (Figure 3a). However, in terms of absolute cell numbers, CD3-high status was most closely associated with large numbers of CD8^+ ^cells (7.8-fold higher in CD3 high versus low). When patients were categorized according to high/low levels of CD8^+ ^and CD4^+ ^TIL, anthracycline-based therapy was beneficial for CD8-high (HR, 0.36; 95% CI, 0.15 to 0.84; *P *= 0.0177), but not for CD8-low patients (HR, 0.74; 95% CI, 0.35 to 1.59; *P *= 0.4408; Figure 3b). In contrast, treatment efficacy showed no association with CD4 status (Figure 3c), suggesting that CD8^+ ^TIL are the dominant TIL phenotype in determining benefit from anthracycline-based therapy. This is consistent with our observation that the CTL effector gene *GZMB *was a strong predictor of therapeutic response in the neoadjuvant cohort. To confirm this, we scored the adjuvant cohort for cells expressing TIA-1, a component of cytolytic granules characteristic of differentiated CTL and natural killer cells [5,22-28]. TIA-1 was present at detectable levels in 58 of 137 assessable cases and correlated strongly with CD8 (Spearman *R *= 0.64; *P *< 0.0001). Among patients treated with anthracycline-based therapy, no recurrences occurred for those with high levels of TIA-1^+ ^TIL (upper quartile or higher), whereas those with low levels of TIA-1 had a DFS rate of only 29.6% (log-rank HR, 0.16; 95% CI, 0.05 to 0.52; *P *= 0.0027; data not shown). Like CD3, TIA-1 had no significant prognostic relevance for CMF-treated patients (log-rank HR = 0.60; 95% CI, 0.21 to 1.71; *P *= 0.3397). These data collectively suggest that cytotoxic T-cells are a key component of the association between TIL and responsiveness to anthracycline-based therapy in ER-negative breast cancer.

### TIL predict anthracycline sensitivity in distinct molecular subtypes of ER-negative breast cancer

Her2 overexpression is frequently observed in ER-negative breast cancer and is a well-known marker of poor prognosis. While trastuzumab use has markedly improved management of Her2-positive breast tumors, standard chemotherapeutic approaches continue to play a role in the treatment of these cancers. Although our second cohort did not contain enough Her2-positive cases treated with anthracyclines to permit rigorous survival analysis, we assessed clinical responses in the Her2-positive subset of the neoadjuvant (EORTC) cohort. Of seven cases classified as TIL-high, six achieved a pCR (85.7%), compared with only eight of 23 TIL-low cases (34.8%; Fisher's exact test: *P *= 0.0309; OR, 11.25; 95% CI, 1.145 to 110.5).

Unlike ER-positive and Her2-positive breast tumors, which are candidates for endocrine and trastuzumab therapy, respectively, no targeted therapies for triple-negative breast tumors (TN; those lacking ER, PR, and Her2 overexpression) currently exist, making these a priority of translational research efforts. We therefore investigated whether our findings also applied to TN tumors. Among cases with documented Her2, ER, and PR status, TN tumors constituted 71% of the neoadjuvant (EORTC) cohort and 61% of the adjuvant (MBTB) cohort. In the neoadjuvant cohort, the predictive capacity of TIL status for anthracycline responsiveness in the TN subset was comparable to that of the entire dataset; the pCR rates of the TIL-high and -low groups were 73.9% and 30.9%, respectively (Fisher's exact test: *P *= 0.0009; OR, 6.333; 95% CI, 2.124 to 18.89). Neither *CD19 *nor *MS4A1 *alone correlated with pCR, whereas *GZMB *did so significantly (*P *= 0.0042). Similarly, in TN tumors of the MBTB adjuvant cohort, anthracycline-based therapy was associated with improved survival in the CD3-high group (log-rank HR, 0.25; 95% CI, 0.09 to 0.66; *P *= 0.0056), but not the CD3-low group (log-rank HR, 0.65; 95% CI, 0.24 to 1.79; *P *= 0.4054; data not shown). Consistent with the neoadjuvant cohort, CD8^+ ^(*P *= 0.0390) and TIA-1^+ ^(*P *= 0.0040) TIL were associated with improved outcomes after anthracycline-based therapy, but not CD4^+ ^or CD20^+ ^TIL. Therefore, TIL (particularly CTL) are associated with sensitivity to anthracycline-based therapy in the clinically challenging triple-negative subset of breast cancer.

## Discussion

We have demonstrated that a subgroup of ER-negative breast cancer defined by high lymphocyte gene expression has a remarkably high rate (74%) of pathologic complete response to neoadjuvant anthracycline-based therapy. We have further shown that the clinical benefit of adjuvant anthracycline-based therapy may be restricted to patients with high numbers of tumor-infiltrating T-cells, especially those of the CD8^+ ^and TIA-1^+ ^subsets. In contrast, TIL show no association with outcome after classic CMF therapy.

Our findings are consistent with several prior reports. In 2008, Hornychová *et al. *[29] observed high levels of intraepithelial CD3^+ ^TIL in pretreatment biopsies of patients who achieved pCR after neoadjuvant therapy with doxorubicin and paclitaxel. Ladoire *et al. *[30] analyzed pre- and post-treatment samples from 56 patients treated with neoadjuvant anthracycline-based therapy and found that high ratios of CD8^+ ^to Foxp3^+ ^TIL (putative regulatory T-cells (Tregs)) in surgical specimens were correlated with a high rate of pCR. Consistent with our own findings, they also reported that, relative to pretreatment levels, TIA-1^+ ^and GzmB^+ ^TIL (CTL) were significantly increased in the posttreatment samples of patients who achieved pCR, but not in those who had residual disease. Finally, Denkert *et al. *[11] reported a significant relationship between TIL (identified by a combination of H&E assessment, IHC for CD3 and CD20, and expression analysis of several TIL genes by PCR) and pathologic response to neoadjuvant anthracycline/taxane therapy in a large group of 1,058 patients, approximately one fourth of whom were ER-negative. As such, based on our data and prior reports, TIL are consistently associated with improved outcomes after anthracycline-based therapy. Intriguingly, in 2001, Demaria *et al. *[31] observed in a small group of breast cancer patients (*n *= 25) treated with neoadjuvant paclitaxel therapy that levels of CD3^+ ^TIL increased in the tumor bed after treatment, and that this correlated with therapeutic response. Although our data imply that TIL are not related to therapeutic responses in patients treated with cisplatin or CMF, the Demaria study nevertheless suggests that the correlation of TIL with treatment response may not be restricted entirely to anthracycline-based therapy.

It is important to note that TIL have been correlated with improved survival in several breast cancer studies to date, the association being observed primarily in high-grade, ER-negative, or Her2-positive lesions [4-10]. Given that many of the patients evaluated in these studies were not systemically treated, TIL may also be a feature of breast cancers with a naturally favorable clinical outcome, which could have implications for the interpretation of the results in our study. This may also be the case for a variety of other cancer types, including malignancies of the colon [32], ovary [33], lung [34], and bladder [35]. In the systemically untreated patients of our long-term follow-up cohort, however, those with high levels of CD3^+ ^or CD8^+ ^TIL had only a modest, nonsignificant survival advantage over those with low TIL levels. It is therefore unlikely that the inherent association between TIL and improved prognosis was a significant underlying feature of our observations with regard to chemotherapy response.

The mechanism underlying the relationship between TIL and anthracycline sensitivity in humans is not currently understood. It is possible that TIL may not be causally related to therapeutic outcomes, and are instead biomarkers of an unknown feature that burdens tumors with anthracycline sensitivity. However, it is equally plausible that TIL directly mediate treatment responses. Indeed, this is supported in preclinical models, discussed here at length.

Four primary explanations for the relationship between anthracyclines and immunity have arisen based on observations in both the laboratory and the clinic [36,37]. One possible scenario is that anthracyclines kill or suppress Tregs in the tumor microenvironment, thus relieving inhibition of the anti-tumor immune response. For example, in the aforementioned study by Ladoire *et al. *[30], Foxp3^+ ^TIL were significantly reduced in the tumor bed after successful neoadjuvant anthracycline-based therapy, whereas their levels were unchanged in patients with residual disease. However, interpretation of this result is difficult because patients in the Ladoire study received a regimen (FEC) containing cyclophosphamide, a drug known to deplete Tregs selectively at low doses (as well as conventional T-cells at high doses) [38-41]. Similarly, many of the drug combinations used to treat patients in our study also included cyclophosphamide. Further studies designed to elucidate the specific effects of anthracyclines on intratumoral Treg populations will be required to determine the validity of this concept.

A second possible mechanism is derived from observations that transient lymphopenia (induced therapeutically via low-dose total-body irradiation or by myelosuppressive drugs such as cyclophosphamide) can enhance immunotherapy in the context of adoptive transfer or vaccination. Through a variety of mechanisms, therapeutic lymphopenia appears to trigger homeostatic processes that favor the proliferation and functionality of anti-tumor effector T-cells, while also ameliorating tumor-induced immunosuppression [42]. However, considering the myelosuppressive properties of cyclophosphamide, this idea does not account for our observation that TIL are associated with outcome after anthracycline-based but not CMF chemotherapy.

A third hypothesis is that chemotherapy can sensitize tumor cells to T-cell-mediated cytotoxicity. For example, Yang and Haluska [43] found that treatment of human melanoma cell lines with either 5-fluorouracil or dacarbazine improved the efficacy of perforin/granzyme-mediated killing by antigen-specific CTL. Similar results have been reported with respect to paclitaxel, cisplatin, and doxorubicin [44]. Cyclophosphamide has also been shown to sensitize mesothelioma cells to CTL killing, although through a TRAIL (TNF-related apoptosis-inducing ligand)-mediated effect [45]. Finally, doxorubicin has been shown to induce Fas expression in breast cancer cell lines, leading to increased susceptibility to Fas ligand-mediated apoptosis [46]. These studies collectively suggest an attractive model involving cytotoxic synergy between chemotherapy and the immune system. Nevertheless, as with the myelosuppression model discussed above, this mechanism would support a relationship between TIL and CMF therapy, which is not observed in our study.

The fourth hypothesis is that anthracyclines, unlike most chemotherapeutic drugs, induce immunogenic tumor cell death and thereby function indirectly as immunostimulatory agents [42]. Casares *et al. *[47] treated murine CT26 colon carcinoma cells with doxorubicin and inoculated the dying cells into syngeneic hosts, which resulted in protection from concurrent or subsequent challenge with live CT26 cells. Notably, this effect was dependent on dendritic cells and CD8^+ ^T-cells and was specific for the CT26 tumor line, indicating an immunologic mechanism. In contrast, cells stimulated to undergo apoptosis by using mitomycin-C, a non-anthracycline DNA cross-linking agent, did not elicit protective immunity. Obeid *et al. *[48] expanded on this work by demonstrating that anthracyclines trigger preapoptotic shuttling of calreticulin to the dying cell surface, where it acts as an opsonin to stimulate phagocytosis and processing by dendritic cells. Compared with a variety of apoptosis-inducing drugs with various mechanisms of action (including staurosporine, tunicamycin, mitomycin-C, and etoposide), anthracyclines induced greater calreticulin surface presentation on target cells, which correlated with the ability to elicit protective immunity. Indeed, blockade or knockdown of calreticulin in anthracycline-treated cells abolished their immunogenicity in mice [48]. Finally, Apetoh *et al. *[49] demonstrated that doxorubicin additionally causes the extracellular release of HMGB1 (high-mobility-group box 1), an endogenous ligand of Toll-like receptor 4 (TLR4). HMGB1 stimulation of dendritic cells was necessary for processing and cross-presentation of antigens from dying tumor cells. The authors further showed that breast cancer patients bearing loss-of-function *TLR4 *alleles had significantly worse prognosis when treated with anthracycline-based chemotherapy [49]. Taken together, mounting evidence indicates that anthracyclines have the rare ability to elicit specific immunity against target cells via stimulation of dendritic cells in a calreticulin and HMGB1/TLR-4-dependent fashion. This may explain the observation that anthracycline-based therapy appears most effective in breast cancer patients with high levels of endogenous tumor immunity, as well as the finding of Ladoire *et al. *[30] that CTL accumulate in the tumor beds of patients treated successfully with neoadjuvant anthracycline-based therapy. One unanswered question is whether anthracycline-based treatments can induce new tumor antigen-specific T-cells (*de novo *immunity) or, alternatively, augment the expansion or functional activity of preexisting tumor-reactive T-cells.

An important confounding feature of our study is the heterogeneous and multivalent chemotherapy used in the assessed cohorts. Although we have emphasized anthracyclines as the unifying components of the treatments we assessed, it is difficult to determine the extent to which anthracyclines are specifically responsible for our observations. For example, many of the patients in this study were treated with regimens involving taxanes, which may also have clinically relevant effects on host immunity [31]. Furthermore, the potentially competing or cooperative effects on tumor immunity that agents such as anthracyclines, cyclophosphamide, and taxanes may possess when administered sequentially or simultaneously are not currently understood. A second caveat worthy of note pertains to the use of gene-expression arrays to infer the presence of TIL. The use of IHC to detect TIL is advantageous for several reasons, including the ability to directly quantify cells that express a given marker, as well as to accurately determine their specific localization within a tissue. This information cannot be acquired by using conventional methods of gene-expression analysis. Thus, although the data presented for the eight-gene TIL signature reflect a relative abundance of TIL, they lack the desirable single-cell resolution that is achievable through IHC.

A further limitation of this study is that the adjuvant cohort did not contain enough anthracycline-treated cases to permit rigorous multivariate analysis (although this was performed in the neoadjuvant cohort). This was largely because ER-negative tumors constitute only ~30% of breast cancer cases, and CMF was the dominant systemic regimen for breast cancer in Canada at the time that this cohort was treated. Our additional validation cohorts are also small for two primary reasons: first, because many publically available datasets are derived from fine-needle aspirates (as opposed to core or open-biopsy specimens), and so may not accurately reflect overall lymphocyte infiltration; and second, because most cohorts contain both ER-positive and ER-negative tumors, with relatively few cases of the latter. Our observations thus warrant validation in large, independent cohorts with long-term follow-up. Ultimately, prospective trials of therapeutic outcome prediction will be required to fully assess the clinical utility of the concepts presented herein.

Our findings indicate that TIL, which are easily detectable with standard IHC, may prove useful for identifying patients who are likely to benefit from anthracycline-based therapy. Moreover, our findings suggest that patients with low TIL levels may benefit most from alternative regimens such as CMF. In addition, our study provides support for the idea of combining anthracycline-based chemotherapy with immunotherapeutic strategies such as cancer vaccines or adoptive T-cell therapy to fully engage the host immune response against breast cancer.

## Conclusions

TIL that express cytotoxic markers are strongly associated with favorable outcome after anthracycline-based treatment of ER-negative breast cancer, including the clinically challenging triple-negative subset. Further studies to develop TIL-based predictive and therapeutic strategies for breast cancer are warranted.

## Abbreviations

CTL: cytotoxic T lymphocyte; DFS: disease-free survival; ER: estrogen receptor alpha; Her2: human epidermal growth factor receptor 2; HR: hazard ratio; IHC: immunohistochemistry; pCR: pathologic complete response; PR: progesterone receptor; RD: residual disease; TIA-1: T-cell-restricted intracellular antigen 1; TIL: tumor-infiltrating lymphocytes; TMA: tissue microarray; Treg: regulatory T cell.

## Competing interests

The authors declare that they have no competing interests.

## Authors' contributions

NRW participated in study design, performed analyses, and drafted/revised the manuscript. KM performed the immunohistochemistry. PTT, NM, and BHN contributed to study design, data interpretation, and manuscript revision. PHW conceived the study, scored IHC tissue sections, and participated in data interpretation and manuscript revision. All authors read and approved the final manuscript.

## Supplementary Material

Additional file 1 **Supplementary Figure S1 and Supplementary Table S1**. Figure S1 displays the associations of the genes from the eight-gene TIL signature with clinical outcome in the four publically available datasets assessed in this study (GEO accession number GSE6861, the primary cohort treated with anthracycline-based therapy; GSE21974 and GSE19615, two additional cohorts of anthracycline-treated breast cancer patients; and GSE18864, a cohort treated with cisplatin). Table S1 displays the clinical characteristics of the MBTB cohort, organized by treatment category. Click here for file
