# Supplementary material for: Tumor-infiltrating lymphocytes predict response to anthracycline-based chemotherapy in estrogen receptor-negative breast cancer
Source: Breast Cancer Res. 2011 Dec 8;13(6):R126. doi: 10.1186/bcr3072 (PMC3326568; doi:10.1186/bcr3072)
Supplement: Additional file 1 — Supplementary Figure S1 and Supplementary Table S1. Figure S1 displays the associations of the genes from the eight-gene TIL signature with clinical outcome in the four publically available datasets assessed in this study (GEO accession number GSE6861, the primary cohort treated with anthracycline-based therapy; GSE21974 and GSE19615, two additional cohorts of anthracycline-treated breast cancer patients; and GSE18864, a cohort treated with cisplatin). Table S1 displays the clinical characteristics of the MBTB cohort, organized by treatment category. [file bcr3072-S1.PDF]

Supplementary Materials

Tumor infiltrating lymphocytes predict response to anthracycline-based chemotherapy in estrogen receptor-negative breast cancer

Nathan R. West, Katy Milne, Pauline T. Truong, Nicol Macpherson, Brad H. Nelson, & Peter H. Watson

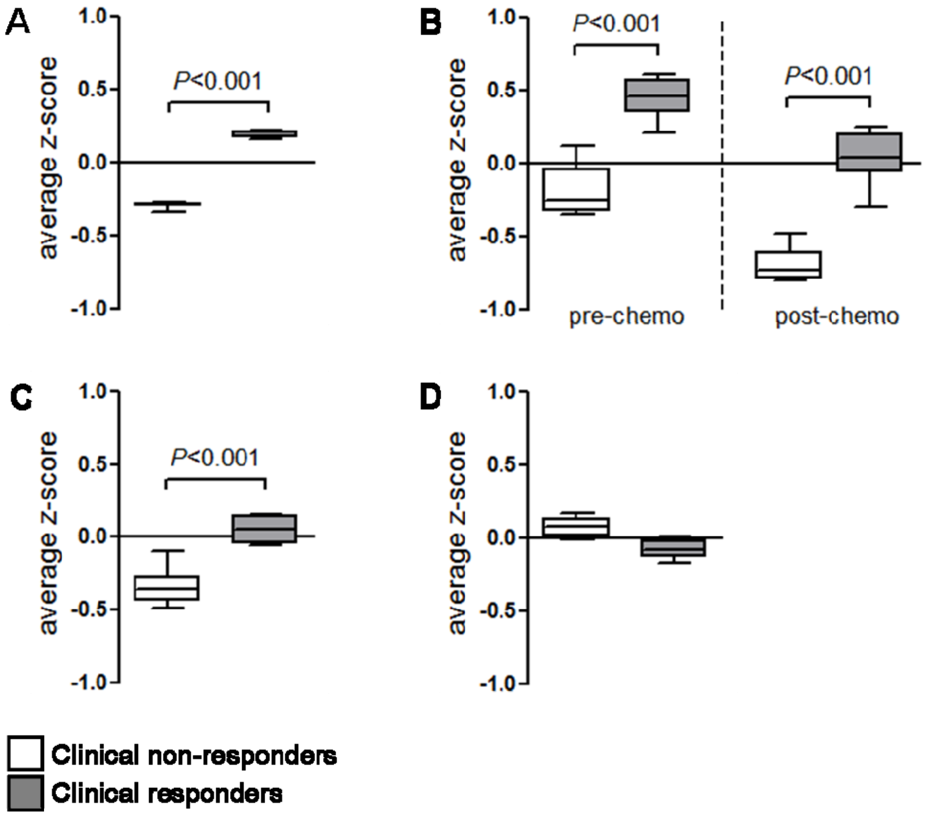

Figure S1. Expression of the 8-gene TIL signature in independent cohorts of estrogen receptor negative breast cancer. The Gene Expression Omnibus accession numbers for the four cohorts are as follows: (**A**) GSE6861 (the primary EORTC/neoadjuvant cohort assessed in this study, included here for comparison with the additional cohorts); (**B**) GSE21974; (**C**) GSE19615; (**D**) GSE18864. Box plots represent the average z-score values of the 8 TIL genes of patients from each clinical response category. For GSE21974, data derived from pre-treatment biopsies and post-treatment surgical specimens are indicated. For full cohort and methodological details, see the Materials and Methods section in the main manuscript.

Table S1. Clinical characteristics of the MBTB cohort, according to treatment received.

		Lumpectomy (*n*=81)		Mastectomy (*n*=174)		Radiotherapy (*n*=100)		Anth. therapy (*n*=57)		CMF therapy (*n*=90)		No chemo (*n*=100)*	
	**Tumor status**	**#**	**%**	**#**	**%**	**#**	**%**	**#**	**%**	**#**	**%**	**#**	**%**
Age at diagnosis	<50 years	32	40%	53	30%	44	44%	33	58%	38	42%	10	10%
	≥50 years	49	60%	119	68%	56	56%	24	42%	52	58%	88	88%
	unknown	0	0%	2	1%	0	0%	0	0%	0	0%	2	2%
Tumor size	≤2 cm	11	14%	22	13%	9	9%	4	7%	11	12%	17	17%
	2< cm ≤5	64	79%	36	21%	74	74%	32	56%	64	71%	60	60%
	>5 cm	2	2%	97	56%	11	11%	9	16%	9	10%	19	19%
	unknown	4	5%	19	11%	6	6%	12	21%	6	7%	4	4%
Nodal status	negative	31	38%	57	33%	36	36%	17	30%	30	33%	37	37%
	positive	34	42%	108	62%	58	58%	37	65%	58	64%	43	43%
	unknown	16	20%	9	5%	6	6%	3	5%	2	2%	20	20%
Grade	1–2	39	48%	81	47%	50	50%	29	51%	37	41%	52	52%
	3	35	43%	84	48%	44	44%	24	42%	45	50%	45	45%
	unknown	7	9%	9	5%	6	6%	4	7%	7	8%	3	3%
ER[†]	negative	81	100%	174	100%	100	100%	100	100%	100	100%	100	100%
	positive	0	0%	0	0%	0	0%	0	0%	0	0%	0	0%
PR[‡]	negative	66	81%	146	84%	77	77%	49	86%	74	82%	81	81%
	positive	15	19%	26	15%	23	23%	8	14%	16	18%	17	17%
	unknown	0	0%	2	1%	0	0%	0	0%	0	0%	2	2%
Her2[§]	negative	46	57%	111	64%	55	55%	41	72%	51	57%	60	60%
	positive	24	30%	34	20%	30	30%	10	18%	20	22%	27	27%
	unknown	11	14%	29	17%	15	15%	6	11%	19	21%	13	13%

*8 patients received chemotherapy, but of an unknown regimen. These patients are not included in the systemic therapy columns.
[†]ER negative status defined as <10 fmol/mg protein (ligand binding assay).
[‡]PR negative status defined as ≤15 fmol/mg protein (ligand binding assay).
[§]Her2 positive status determined by an IHC score of 3.
